# Prescribing of FDA-approved and compounded hormone therapy differs by specialty

**DOI:** 10.1097/GME.0000000000000683

**Published:** 2016-10-03

**Authors:** Ginger D. Constantine, David F. Archer, Shelli Graham, Brian A. Bernick, Sebastian Mirkin

**Affiliations:** 1 EndoRheum Consultants LLC, Malvern, PA; 2 Clinical Research Center, Department of Obstetrics and Gynecology, Eastern Virginia Medical School, Norfolk, VA; 3 TherapeuticsMD, Inc, Boca Raton, FL.

**Keywords:** Compounded hormone therapy, Estrogen therapy, FDA-approved hormones, Menopause, Progesterone

## Abstract

**Objective:**

To determine the prescribing patterns of general practitioners (GPs), obstetrician/gynecologists (OB/GYNs), and wellness physicians (WPs) of menopausal hormone therapy (HT) for both compounded (CHT) and Food and Drug Administration (FDA)-approved products, using a survey of US physicians.

**Methods:**

Nine thousand one US physicians were invited to participate in a survey to report on their HT-prescribing patterns. Physicians were eligible if they prescribed HT for at least six patients per month.

**Results:**

The survey was completed by 440 eligible physicians (893 responded of 9,001 invited) including 171 GPs, 170 OB/GYNs, and 84 WPs. Physicians prescribed HT for 15% to 30% of their female patients, with WPs numerically most likely to prescribe HT. Menopausal symptoms were the leading reason for HT prescriptions among all specialties. WPs seemed more likely to prescribe HT for general/cardiovascular health (28%), and for shorter durations, than other specialties. WPs prescribed proportionally more compounded (vs FDA-approved) estrogens/progestogens than GPs or OB/GYNs, but OB/GYNs seemed to prescribe more compounded dehydroepiandrosterone and testosterone (prescribed alone) than did others. OB/GYNs seemed least likely to consider CHT being more safe or effective than FDA-approved HT. Symptom relief was the main determinant of efficacy for all specialties; WPs also used blood (61%) or saliva testing (25%) for dose adjustment.

**Conclusions:**

Although all physician specialties surveyed prescribed HT, differences in prescribing CHT versus FDA-approved formulations by medical specialty/practice seemed to exist. Of those surveyed, OB/GYNs and GPs prescribed proportionally more FDA-approved HT, whereas WPs, similarly, prescribed more CHT. More discussion is needed concerning physicians’ decisions to prescribe CHT versus FDA-approved formulations.

## Abstract 2

Supplemental Digital Content is available in the text

Menopausal symptoms are common and bothersome to many women.^1,2^ Hormone therapy (HT) was widely used through the 1990s for many indications and to treat menopausal symptoms, but use fell dramatically after the publication of the results from the Women's Health Initiative (WHI) trials in 2002.^3^ Physicians’ attitudes regarding HT reflected those of their patients’ after the release of the WHI information, and prescribing practices shifted.^4,5^ In a survey of US Midwestern healthcare practitioners (physicians and nurse practitioners practicing gynecology, family medicine, and internal medicine) published in 2007, 74% of practitioners responded to the WHI findings by prescribing lower-dose products, and 73% recommended reducing duration of treatment to patients.^4^

The gap between a need for effective menopausal treatment and available therapies with a perceived acceptable risk/benefit ratio vastly expanded the use of non-Food and Drug Administration (FDA)-approved compounded hormone therapy (CHT),^6,7^ which has been marketed as safer than HT and has been promoted as a superior alternative to HT.^6,8-10^ Compounded hormones are not FDA-approved^11^ and are not required to demonstrate safety and efficacy through the rigorous clinical trials required for HT.^12,13^ Although the FDA does maintain some oversight of compounding facilities, their authority is limited and varies by state.^11,13^ Thus, CHT products lack rigorous efficacy data and safety validation related to quality, purity, and potency.^7,14,15^ In addtion, compounded drugs are not required to carry a package insert, outlining the risks associated with HT.^16^ Consequently, many physicians and patients are unaware of the risks associated with CHT, and confusion about the differences between CHT and FDA-approved HT is common among consumers and physicians alike.^8,10,17^

Quantifying the size of the CHT market is difficult owing to the lack of FDA oversight and resultant absence of prescription tracking, but recent estimates indicate that the market size might be substantial. Data from recent surveys suggest that CHT users represent approximately 34% to 60% of the current users of HT (2-3 million women).^17,18^ Younger CHT users (age 40-49 y) represent an even larger percentage of HT users (41% of women who have ever used HT), suggesting that women more recently initiating therapy have a higher likelihood of choosing CHT options.^18^

Little is known about physicians’ attitudes and prescribing patterns of HT, particularly CHT. The objective of this survey was to assess physicians’ prescribing practices of HT, in particular CHT, among US physicians of different specialties. The survey of physicians reported here is the third in a series of three reports on the results of surveys of consumers,^17^ compounding pharmacists,^19^ and physicians, designed to gain a clearer picture of the size of the CHT market and the reasons behind its growth.

## METHODS

US physicians (n = 9,001) were invited to participate in an online survey (Appendix A, Supplemental Digital Content 1) by Rose Research, a market research company. Physicians were recruited from an IRB-approved panel source, Global Market Insite, Inc. (GMI), a global online sample provider. To be eligible to complete the survey, physicians had to currently prescribe HT for at least six patients per month. Participants signed a formal confidentiality agreement and were protected by the privacy policy of Global Market Insite, Inc. The survey consisted of questions addressing HT-prescribing practices including CHT. Surveys were conducted online between May and July of 2014. Physicians were compensated between $25 and $35 in exchange for their time used to complete the survey.

The physicians were categorized as a general practitioner (GP) if their primary area of specialty was reported as “internal medicine/family practice”; an obstetrician/gynecologists (OB/GYN) if their primary specialty was identified as “obstetrics/gynecology”; a wellness physician (WP) if their specialty or practice was indicated as “anti-aging/wellness or regenerative care” or they were not obstetricians, gynecologists, or endocrinologists but said they saw at least 25% of their female patients for “hormone replacement therapy/hormone therapy (counseling and treatment management)”; or as an endocrinologist if their primary specialty was “endocrinology.”

Results are reported as a descriptive analysis.

## RESULTS

### Response rates and physician characteristic*s*

Of 9,001 candidate physicians invited to participate, 893 (10%) responded. Four hundred forty of the respondents (49%) were eligible and completed the survey; 171 were GPs, 170 were OB/GYNs (118 who saw ≥20% of their patients for “obstetrics” and 52 who saw <20% of their patients for “obstetrics”), and 84 were WPs. General practitioners saw an average of 78 female patients per month, whereas OB/GYNs saw 110 and WPs saw 84. Those who did not qualify either did not meet the inclusion criterion of prescribing HT to at least six female patients per month or the specialty group with which they identified did not respond in large enough numbers to conduct meaningful comparisons. Fifteen endocrinologists completed the survey, but the data from this group were not included in the analysis owing to the small sample size. Overall responses of endocrinologists were similar to those of OB/GYNs. Responders were broadly represented geographically, representing 48 states and the District of Columbia (Appendix B, Supplemental Digital Content 1).

### HT-prescribing patterns

**FIG. 1:**
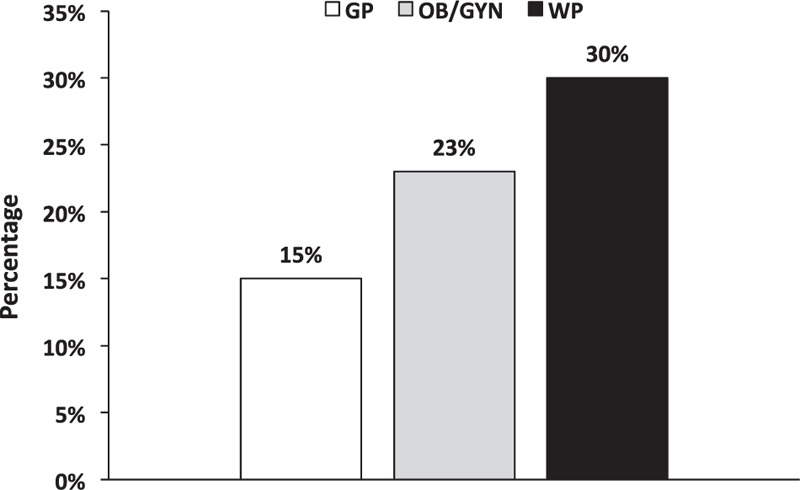
Percentage of patients that were prescribed hormone therapy (FDA-approved and CHT) by specialty. The mean number of female patients per month were: GP, 78; OB/GYN, 111; WP, 84. CHT, compounded hormone therapy; FDA, Food and Drug Administration; GP, general practitioners; OB/GYN, obstetrician/gynecologists; WP, wellness physician.

**FIG. 2:**
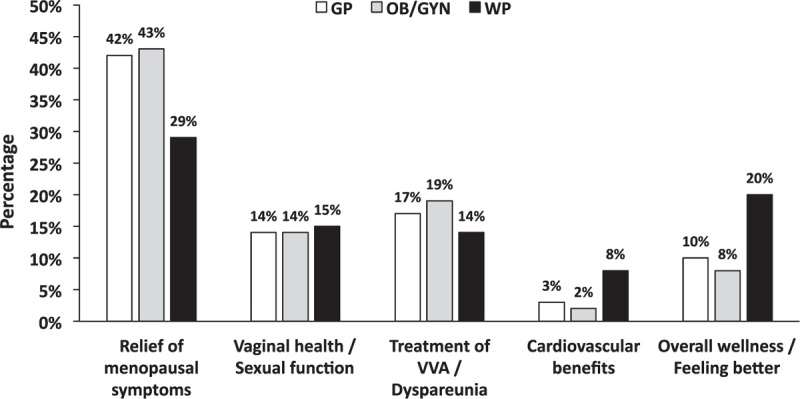
Primary reason HT was prescribed (FDA-approved and CHT) by specialty. The mean number of female patients per month were: GP, 78; OB/GYN, 111; WP, 84. CHT, compounded hormone therapy; FDA, Food and Drug Administration; GP, general practitioners; OB/GYN, obstetrician/gynecologists; WP, wellness physician.

WPs and OB/GYNs prescribed HT to proportionally more women in general compared with GPs (Fig. 1). “Relief of menopausal symptoms” was the leading reason for HT prescription among all specialties (29%-43% of the time, Fig. 2). Physicians also prescribed HT for “treatment of VVA/dyspareunia” (14%-19% of the time) and for “vaginal health/sexual function” (14%-15% of the time, Fig. 2). WPs prescribed HT for “cardiovascular benefits” or for “overall wellness/feeling better” 28% of the time (8% and 20%, respectively), whereas GPs prescribed HT for these indications 13% of the time (3% and 10%, respectively), and OB/GYNs 10% of the time (2% and 8%, respectively, Fig. 2).

**FIG. 3:**
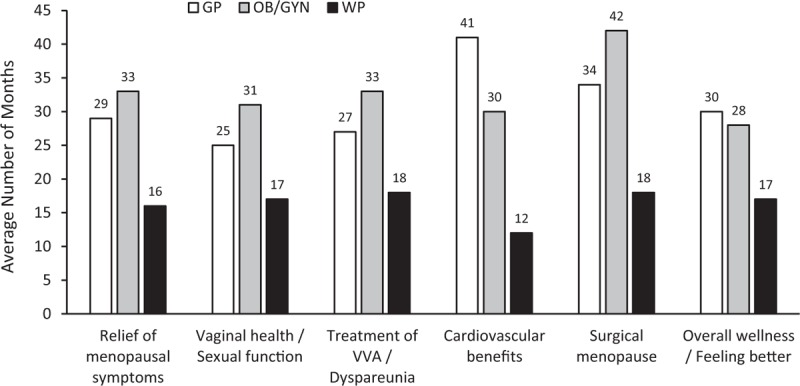
Average number of months HT was prescribed (FDA-approved and CHT) by reasons for prescription and by specialty. The mean number of female patients per month were: GP, 78; OB/GYN, 111; WP, 84. CHT, compounded hormone therapy; FDA, Food and Drug Administration; GP, general practitioners; OB/GYN, obstetrician/gynecologists; VVA, vulvovaginal atrophy; WP, wellness physician.

GPs and OB/GYNs seemed to prescribe HT for longer durations than WPs, and the length of treatment varied depending on the reason for prescription, ranging from 12 to 42 months on average (Fig. 3). OB/GYNs tended to prescribe HT for the longest durations overall. Numerically, the longest duration of prescription for OB/GYNs was for women who had undergone surgical menopause (average of 42 mo), whereas the longest duration of prescription for GPs was for cardiovascular benefits (average of 41 mo).

**FIG. 4:**
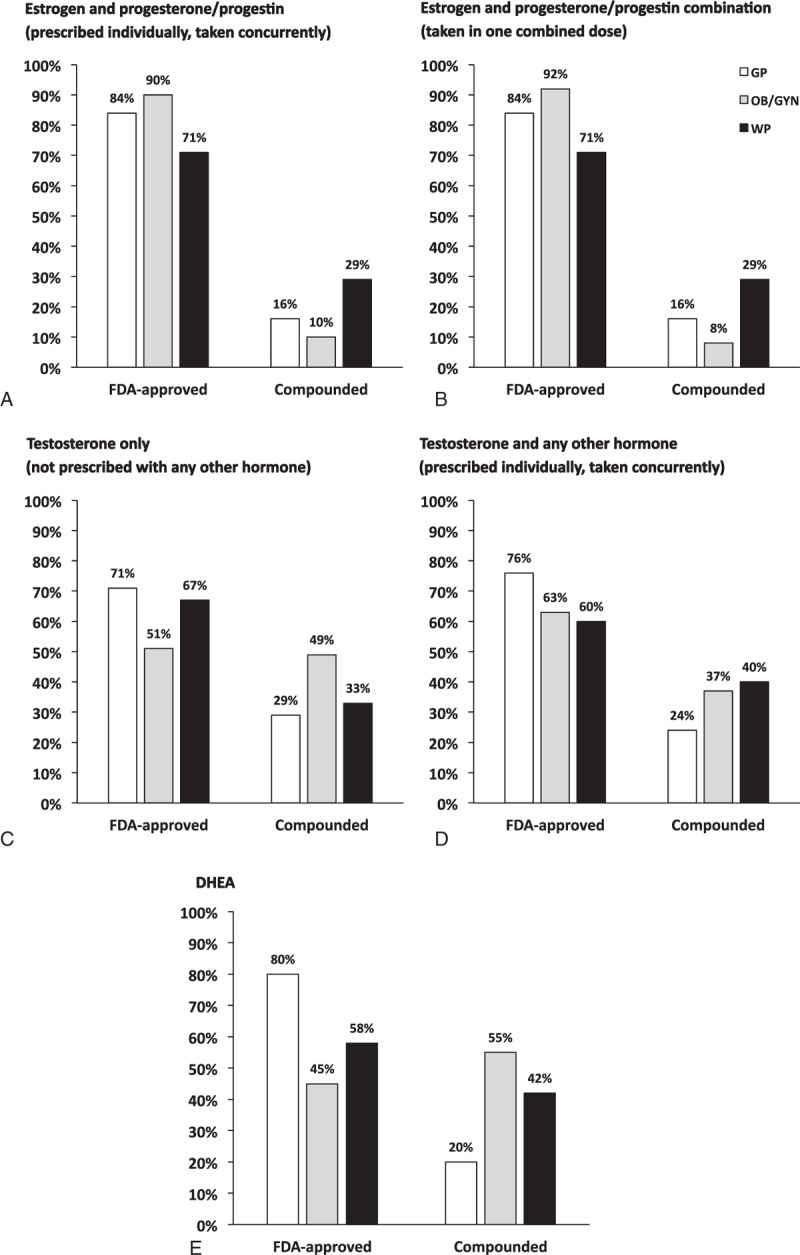
FDA-approved versus compounded HT prescriptions for individual types of hormones by specialty. The mean number of female patients per month were: GP, 78; OB/GYN, 111; WP, 84. DHEA, dehydroepiandrosterone; FDA, Food and Drug Administration; GP, general practitioners; HT, hormone therapy; OB/GYN, obstetrician/gynecologists; WP, wellness physician.

Overall, physicians from all specialties prescribed between 8% and 55% of the reported products as compounded formulations depending on physician's specialty and the hormone prescribed (Fig. 4A-E). Wellness physicians prescribed a seemingly higher percentage of compounded estrogen and progesterone (either prescribed individually and taken concurrently [29%], or prescribed as a combined formulation [29%]) than other specialties. GPs prescribed compounded estrogen and progesterone individually and taken concurrently to 16% of their patients, and compounded estrogen and progesterone in a combined dose to another 16%, whereas OB/GYNs prescribed these combinations to 10% and 8% of their patients, respectively (Fig. 4A-B).

Rates of prescribing compounded dehydroepiandrosterone (DHEA) (55%) and testosterone alone (49%) were numerically greatest for OB/GYNs. When androgens were combined with other hormones, WPs and OB/GYNs, however, seemed to prescribe at similar rates (40% and 37%, respectively; Fig. 4C-E). Although there is no FDA-approved formulation of DHEA or no approved formulation of testosterone for women, some physicians from all specialties reported that they prescribed greater or similar numbers of FDA-approved formulations of both testosterone and DHEA to their female patients than compounded formulations. GPs reported that they prescribed FDA-approved formulations of testosterone alone, testosterone with other hormones, or DHEA to 71%, 76%, and 80% of their patients, respectively. OB/GYNs stated that they prescribed these formulations to 51%, 63%, and 45% of their patients, respectively, and WPs said that 67%, 60%, and 58% of their patients received these prescriptions, respectively.

**FIG. 5:**
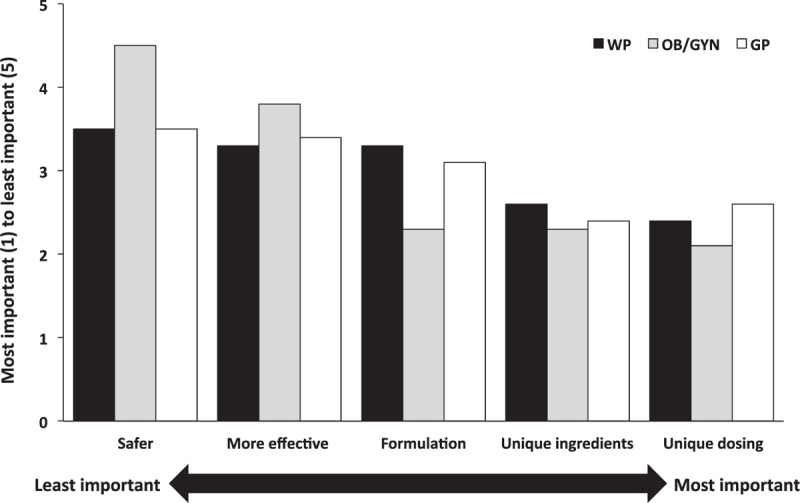
Reasons for prescribing CHT over FDA-approved HT by specialty, ranked by importance. The mean number of female patients per month were: GP, 78; OB/GYN, 111; WP, 84. CHT, compounded hormone therapy; FDA, Food and Drug Administration; GP, general practitioners; OB/GYN, obstetrician/gynecologists; WP, wellness physician.

The reasons ranked most important for prescribing CHT instead of FDA-approved HT were that CHT provided unique dosing and ingredients; however, rankings of reasons seemed to differ slightly by specialty, with OB/GYNs seemingly the least likely to consider CHT more safe or effective than FDA-approved HT (Fig. 5).

**FIG. 6:**
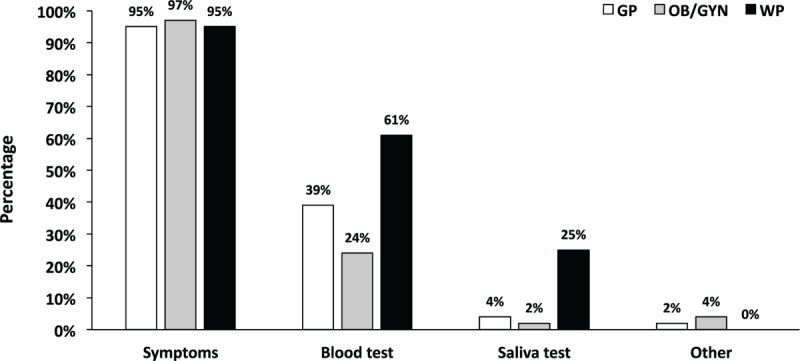
Methods used to monitor HT effectiveness and/or make dose modifications for patients on HT. The mean number of female patients per month were: GP, 78; OB/GYN, 111; WP, 84. FDA, Food and Drug Administration; GP, general practitioners; HT, hormone therapy; OB/GYN, obstetrician/gynecologists; WP, wellness physician.

Physicians from all the three specialties predominately monitored efficacy and/or made dose modifications for their patients taking HT by evaluating symptom relief, although WPs also commonly conducted blood tests (61% of patients) and were more than five times as likely as others to use saliva testing (25% of patients; Fig. 6).

## DISCUSSION

Physicians from all specialties in this survey prescribed HT (both FDA-approved and compounded) to their female patients, but WPs seemed to differ from GPs and OB/GYNs in the percentage of patients prescribed HT, reasons for prescribing hormones, types of compounded hormones prescribed, duration of prescription, and methods used to monitor HT effectiveness.

The reasons for prescribing HT differed numerically among medical specialties. The primary reason for all specialties to prescribe HT was relief of menopausal symptoms, but WPs also prescribed HT for “overall wellness/feeling better” and for “cardiovascular benefits” more than twice as often as GPs or OB/GYNs (Fig. 2). Although only a small percentage of GPs and OB/GYNs prescribed HT for cardiovascular health, the average duration of treatment seemed longer than that of WPs (Fig. 3). Prescribing HT for cardiovascular benefits or other long-term health benefits contradicts current consensus statements from medical societies and the package inserts of HT products.^20-25^

WPs reported prescribing hormones (both FDA-approved and compounded) to their female patients twice as much as GPs and almost one-third more than OB/GYNs (Fig. 1); they also prescribed compounded drugs, which are not monitored by the FDA, to proportionally more of their patients than their GP and OB/GYN counterparts (Fig. 4). Unique dosing and ingredients were commonly reported as important reasons for prescribing CHT (rather than FDA-approved HT), especially by OB/GYNs (Fig. 5). This may reflect an unmet need for well-studied, regulated, alternative regimens to these natural products that do not exist currently on the market.

OB/GYNs seemed more likely to report prescribing compounded testosterone and compounded DHEA than GPs or WPs (Fig. 4C, E). Despite the fact that there is no FDA-approved testosterone for women or DHEA for women or men, physicians of all specialties erroneously thought that DHEA and testosterone were FDA-approved, as evidenced by the positive responses regarding prescribing “FDA-approved formulations” of both testosterone and DHEA to female patients (Fig. 4C-E). One possible explanation for this misunderstanding is that testosterone is FDA-approved for use in men only, but is routinely used at lower doses for women. The lack of knowledge regarding FDA approval status and regulations of such products limits prescribers’ ability to adequately inform their patients of the limitations and risks of these products as compounded. Therefore, many women do not understand the differences between HT and CHT.^8,10,17^ In a survey of 855 peri- and postmenopausal women, only 14% reported that they knew that CHT was not FDA-approved; 76% said “not sure” and 10% said they believed that CHT was FDA-approved.^17^ Iftikhar et al^8^ reported that 67% of 184 women seeking consultation for menopausal concerns believed that CHT was safer than FDA-approved HT, and that 77% of the 31 women who were users of CHT believed the same. Qualitative interviews of 25 postmenopausal women and 31 antiaging clinicians also showed that respondents (both patients and clinicians) valued compounded hormones over FDA-approved options because they perceived them to be “natural” and thus safer.^10^ Confusion on the part of physicians may contribute to women's lack of knowledge about treatment options.^10^ Physicians are a critical source of information regarding potential menopausal symptom treatment options for their patients, and therefore should educate themselves about CHT versus FDA-approved HT.

Compounded estrogens (with or without progesterone), as opposed to FDA-approved versions, were proportionally more commonly prescribed by WPs than by GPs and OB/GYNs (Fig. 4A-B). Common compounded estrogen formulations may contain estradiol, estrone, or estriol, alone or in some combination.^16,26^ Estriol, a metabolite of estradiol and estrone, is a weak estrogen^16,27^ and has limited bioavailability because of its rapid conjugation after oral administration (up to 98% within 6 hours^28^) and low relative binding affinities for estrogen receptors α and β.^16,29^ Estriol is commonly compounded in combination with dosages of estradiol that are high enough to achieve biological effect alone, and thus any perceived efficacy may be due to the estradiol in the formulation.^16,29^ The high doses of estriol, however, required to achieve any biological effect might increase the risk for side effects, thus increasing the risks of endometrial cancer, and venous thromboembolism.^7,30^ Estriol is not FDA-approved,^31^ and can only be compounded provided that a new drug application is filed for use in compounding.^32^ There is a United States Pharmacopeia monograph for estriol, however, which technically allows compounders to use it as an active ingredient in compounding.^32^ Although estriol and estrone are not FDA-approved and therefore only available in CHT products, estradiol is available in multiple doses and FDA-approved formulations.^26^ Why compounded estradiol would be chosen instead of the FDA-approved version is unclear, although the unique dosing available with CHT may be an important reason.

Most of the physicians monitored HT effectiveness and made dose modifications based on symptom relief (Fig. 6), which follows The North American Menopause Society's recommendations^31^ and guidance from the FDA.^12^ Twenty-five percent of WPs used saliva tests to monitor HT effectiveness and 61% used blood tests (Fig. 6), despite the lack of scientific evidence demonstrating a relationship between hormone levels in blood or saliva and menopausal symptoms.^27^ Menopause status can be determined by blood and saliva tests, but the FDA specifies that these tests have not been proven appropriate for use in HT dosage adjustment.^33^ In a review of CHT, Boothby et al^16^ concluded that large interassay and within-patient variability and poor reproducibility of salivary assays, along with a lack of data regarding the pharmacokinetics, pharmacodynamics, volume of distribution, protein binding, route of elimination, and other features of CHT, renders salivary testing clinically inadequate for menopausal hormones. Moreover, no peer-reviewed studies show correlations between salivary or serum hormone levels and menopausal symptoms.^27^ The American College of Obstetricians and Gynecologists points out that salivary hormone level testing for individualization of therapy is not useful for steroid hormones,^7^ and the Endocrine Society's Position Statement on Bioidentical Hormones states that claims that saliva tests can provide the information necessary to customize hormone doses are not supported by scientific data.^34^

The size of the CHT market has been difficult to estimate because CHT prescriptions are not tracked. This report is the third in a series of surveys designed to elucidate the scope of the CHT market in the United States.^17,19^ The first was a report using prescription claims for FDA-approved HT and US Census data to extrapolate data from surveys of consumers to estimate the number of women using CHT annually at up to 2.5 million, representing 28% to 68% of all HT prescriptions.^17^ The second report, using a survey of pharmacists, National Community Pharmacists Association data, and IBISWorld data, concluded that approximately 26 to 33 million CHT prescriptions are filled annually at a cost of between $1.3 and $1.6 billion.^19^ A recent assessment of the rate of CHT use, from a survey reported by The North American Menopause Society, corresponds with these estimates, placing CHT at approximately 34% of the HT market.^18^

The primary limitations of this report include the small sample size of each provider type and the limited number of questions in the survey regarding CHT-prescribing patterns. The sample was limited to physicians who prescribe HT to at least six female patients per month and thus may not represent professional prescription patterns in general. Our market research, however, shows that this group accounts for approximately 75% of the total volume of FDA-approved HT prescriptions, and thus reasonably represents an active HT-prescribing sample. Potential bias is associated with survey questionnaires^35^ or self-reporting.^36^ The payment of a stipend to physician patients might also introduce bias; however, the stipend was typical for specialty survey patients.^37^ Those physicians who responded to the survey possibly differed in characteristics from those who chose not to participate. As demographic data for nonrespondents was not available, comparisons could not be made between respondents and nonrespondents. Owing to the limitations of the study, the results may not be generalizable to each type of physician and should be interpreted with caution. The strengths of this survey were that it was administered by an experienced market research company with large geographical breadth and rigorous quality management,^37^ and the 10% response rate was not unexpected, as it is typical for online surveys.^38^

## CONCLUSIONS

Both FDA-approved HT and CHT were prescribed across all of the specialties assessed in this survey, but there were differences by specialty in prescribing practices for HT. WPs were proportionally more likely to prescribe CHT, and to prescribe HT for cardiovascular benefits and general well-being. OB/GYNs were proportionally less likely to believe that compounding was safer; however, the major reason that OB/GYNs prescribed CHT over FDA-approved HT was unique dosing or ingredients. Given the findings of the lack of awareness of the differences between CHT and HT demonstrated by some physicians prescribing CHT and the number of women who are taking CHT in the United States, this report underscores the need for more discussion about the prescribing patterns, safety, and efficacy of CHT formulations.

## Supplementary Material

Supplemental Digital Content
