# Supplementary material for: Prescribing of FDA-approved and compounded hormone therapy differs by specialty
Source: Menopause. 2016 Oct 3;23(10):1075–82. doi: 10.1097/GME.0000000000000683 (PMC5028143; doi:10.1097/GME.0000000000000683)
Supplement: Supplemental Digital Content [file menop-23-1075-s001.docx]

**Appendix A. Survey of physicians prescribing hormone therapy.**

Q1a. Please check the one that best describes the primary area of specialty that you practice. (CHECK ONE ONLY)

Q1b. And, please indicate the one that best describes the secondary area (if any) of specialty that you practice. (CHECK ONE ONLY)

|  | Q1a | Q1b |
| --- | --- | --- |
| Obstetrics and gynecologist |  |  |
| Anti-aging and wellness/Regenerative Medicine * |  |  |
| Internal medicine/Family practice |  |  |
| Psychiatry* |  |  |
| Cardiology* |  |  |
| Cosmetic/Plastic Surgery* |  |  |
| Dermatology* |  |  |
| Endocrinology* |  |  |
| Other _________________ |  |  |
| None |  |  |

Q2a. Are you board certified?

| Yes |  |
| --- | --- |
| No |  |

Q2b. In which area(s) are you board certified? (CHECK ALL THAT APPLY)

| Anti-aging/Wellness or Regenerative Care |  |
| --- | --- |
| Endocrinology |  |
| Family Medicine |  |
| General Preventive Medicine |  |
| Internal Medicine |  |
| Obstetrics & Gynecology |  |
| Plastic Surgery |  |
| Other _________________ |  |
| None of the above |  |

Q3a. What percentage of your patients do you see for the following reasons…?

| General Health (i.e. acute care for cold & flu, injury, emergency care, annual physicals – non-gynecological) |  |
| --- | --- |
| Chronic Disease – screening, counseling and treatment management (ie. diabetes, high blood pressure, thyroid disease, etc.) |  |
| Cardiology |  |
| Cosmetic Procedures |  |
| Dermatological exams and procedures |  |
| Obstetrics |  |
| Gynecological exams and procedures |  |
| Hormone Replacement Therapy/Hormone Therapy (counseling and treatment management) |  |
| Anti-aging and Regenerative Medicine |  |
| Other _________________ |  |

Q3b. In which state do you currently work?

_________________

Q4. Do you prescribe hormone therapy for any of your patients?

| Yes |  |
| --- | --- |
| No |  |

Q5. On average how many patients per month do you prescribe hormone therapy for?

| 1 to 5 |  |
| --- | --- |
| 6 to 10 |  |
| 11 to 25 |  |
| 26 or more |  |

Q6. Which of the following, if any, do you prescribe for hormone therapy? Please indicate by a percentage.

|  | Men | Women |
| --- | --- | --- |
| Progesterone/Progestin only (not prescribed with any other hormone) |  |  |
| Estrogen only (not prescribed with any other hormone) |  |  |
| Testosterone only (not prescribed with any other hormone) |  |  |
| Estrogen and progesterone/progestin in combination (taken in one combined dose) |  |  |
| Testosterone in combination with any other hormone (taken in one combined dose) |  |  |
| Estrogen and progesterone (prescribed individually, but taken concurrently) |  |  |
| Testosterone and any other hormone (prescribed individually, but taken concurrently) |  |  |
| Growth hormone |  |  |
| DHEA |  |  |
| Other _________________ |  |  |
| **TOTAL** | **100%** | **100%** |
| None of the above |  |  |

Q7. On average, how many patients do you see in a week?

Male ________________ Female* ________________

Q8. What percentage of your female patients do you prescribe hormone therapy (excluding contraceptive therapy) ?

________________%*

Q9. By percentage indicate the primary reason that you prescribe hormone therapy.

| Relief of menopausal symptoms |  |
| --- | --- |
| Vaginal health/Sexual function |  |
| Treatment of vulvar vaginal atrophy/dyspareunia |  |
| Cardiovascular benefits |  |
| Surgical Menopause |  |
| Overall wellness/Feeling better (energy, mood, cosmetic benefits, weight) |  |
| Other _________________ |  |

Q10. How do you monitor the effectiveness and/or make dose modifications for your patients on hormone therapy? (CHECK ALL THAT APPLY)

| Symptoms |  |
| --- | --- |
| Blood test |  |
| Saliva test |  |
| Other _________________ |  |

Q11. What is the average number of months of hormone therapy you prescribe to your patients by indication?

| Relief of menopausal symptoms |  |
| --- | --- |
| Vaginal health/Sexual function |  |
| Treatment of vulvar vaginal atrophy/dyspareunia |  |
| Cardiovascular benefits |  |
| Surgical Menopause |  |
| Overall wellness/Feeling better (energy, mood, cosmetic benefits, weight) |  |
| Other _________________ |  |

Q12. What percentage of your patients that you prescribe hormone therapy to:

|  | % |
| --- | --- |
| Come in requesting hormone therapy before any consultation |  |
| Were not familiar with or reluctant to use hormone therapy and agreed to based on your recommendation |  |
| Were familiar with hormone therapy and based on your consultation, hormone therapy was prescribed |  |

Q13a. What percentage of your patients fill their prescription at the following

| Over the internet from an internet pharmacy |  |
| --- | --- |
| National/regional chain pharmacy or supermarket (i.e. CVS, Walgreens, Walmart, Safeway, etc.) |  |
| Local pharmacy/compounding pharmacy |  |
| Physician’s office |  |
| Other _________________ |  |

For the following question, there are two types of hormone therapy prescribed for women:

- FDA approved hormone therapy in standard doses (i.e. Premarin or Prempro, Estrace, Vivelle-Dot, Climara, etc.) and/or
- Compounded hormone therapy individually prepared for the patient (i.e. BiEst, TriEst, Estriol, etc.)

Q13b. Please indicate by percentage the type(s) of hormone therapy you prescribe for women.

|  | % |
| --- | --- |
| FDA Approved Hormone Therapy |  |
| Compounded Hormone Therapy |  |
| Total | 100% |

Q14a. Please indicate which hormone therapy products you currently prescribe and, in what proportion do you prescribe each?

|  | Compounded Hormone Therapy | FDA-Approved Hormone Therapy | Total |
| --- | --- | --- | --- |
| Progesterone/Progestin only (not prescribed with any other hormone) |  |  | 100% |
| Estrogen only (not prescribed with any other hormone) |  |  | 100% |
| Testosterone only (not prescribed with any other hormone) |  |  | 100% |
| Estrogen and progesterone/progestin in combination (taken in one combined dose) |  |  | 100% |
| Testosterone in combination with any other hormone (taken in one combined dose) |  |  | 100% |
| Estrogen and progesterone (prescribed individually, but taken concurrently) |  |  | 100% |
| Testosterone and any other hormone (prescribed individually, but taken concurrently) |  |  | 100% |
| Growth hormone |  |  | 100% |
| DHEA |  |  | 100% |
| Other _________________ |  |  | 100% |

Q14b. Please rank the reasons listed below in terms of the reasons why you prescribe compounded hormone therapy. Please use a “1” for the most important reason, a “2” for the second most important reason, etc. (RANK ALL 5 REASONS)

|  | RANK |
| --- | --- |
| Necessity for unique dosing not available in an FDA approved product |  |
| Safer than an FDA approved product |  |
| More effective than an FDA approved product |  |
| Necessity of the combination of unique ingredients not available in an FDA approved product |  |
| Form (product type – gel, lotion, suppository, etc.) not available in an FDA approved product |  |

Q15. What percentage of hormone therapy that you prescribe is…?

|  | % |
| --- | --- |
| Oral |  |
| Vaginal |  |
| Transdermal |  |
| Pellet |  |
| Injection |  |
| Sublingual |  |
| Other _________________ |  |

Q16. In development is a drug candidate that is a novel combination of bio-identical 17ß-estradiol and natural bio-identical progesterone that may provide a safer and more effective alternative compared to current hormone therapies.

If this bio-identical combination of estradiol and natural progesterone product were available in a FDA-approved single capsule with better bioavailability allowing for therapeutic benefits at lower doses, how likely would you be to prescribe it in women with an intact uterus over the current options available in the market today? (CHECK ONE ONLY)

| Extremely likely |  |
| --- | --- |
| Very likely |  |
| Somewhat likely |  |
| Not very likely |  |
| Not at all likely |  |

Q17. How unique do you consider this hormone product compared to other FDA-approved products currently on the market? (CHECK ONE ONLY)

| Extremely unique |  |
| --- | --- |
| Very unique |  |
| Somewhat unique |  |
| Not very unique |  |
| Not at all unique |  |

Q18. Using a scale from 5 to 1 where a 5 means extremely believable and a 1 means not believable at all, how believable is it that a product like this can be approved by the FDA and brought to market? (CHECK ONE ONLY)

| 5 – Extremely believable | 4 | 3 | 2 | 1 – Not believable at all |
| --- | --- | --- | --- | --- |

Q19a. And, would you prescribe this hormone product as a replacement or in addition to the products(s) you currently prescribe? (CHECK ONE ONLY)

| As a replacement |  |
| --- | --- |
| In addition to |  |

Q19b. And, what would be the primary reason you would use this product as a replacement to the product(s) you currently prescribe? Please be specific.

Q20. When thinking about the hormone product described above, please rank the following terms in the order that would most closely describe the product. Please use a “1” for the one that best describes this product, a “2” for the one that next best describes this product, etc. (RANK)

| Bio-Identical |  |
| --- | --- |
| Body-Identical |  |
| Natural |  |
| Systemic |  |
| Synthetic |  |
| Other _________________ |  |

Q21. Finally, which of the following terms do you think should be used as the best sales strategy for this hormone product? (CHECK ONE ONLY)

| Bio-Identical |  |
| --- | --- |
| Body-Identical |  |
| Natural |  |
| Systemic |  |
| Synthetic |  |
| Other _________________ |  |

Q22. Please rank in order of preference of the following hormone therapy regimens for those patients requiring combination estrogen and progesterone therapy (1 represents the most preferred therapy). (RANK TOP 5)

| A NEW FDA-approved oral bio-identical Estradiol with a NEW FDA-approved oral bio-identical/natural progesterone all in a single capsule |  |
| --- | --- |
| Existing FDA-approved oral Estradiol with FDA-approved oral natural progesterone (i.e. Estrace + Prometrium) |  |
| Existing FDA-approved oral Estradiol plus norethindrone acetate or medroxyprogesterone acetate |  |
| Conjugated estrogens plus norethindrone acetate or medroxyprogesterone acetate or progesterone |  |
| Activella (Estradiol / Norethindrone) |  |
| Angeliq (Estradiol / Drospirenone) |  |
| FemHRT (Ethinyl Estradiol / Norethindrone) |  |
| Prempro / Premphase |  |
| Combipatch (Estradiol + Norethindrone) |  |
| **DUAVEE**™ (conjugated estrogens/bazedoxifene) |  |
| FDA-approved Estradiol patch plus FDA-approved oral progesterone |  |
| FDA-approved Estradiol patch plus norethindrone acetate or medroxyprogesterone acetate |  |
| FDA-approved Estradiol patch plus compounded progesterone cream |  |
| Compounded HRT oral combination |  |
| Compounded HRT creams |  |
| Compounded HRT oral + cream |  |
| Compounded HRT injection |  |
| Other _________________ |  |

Thank you – that was our last question. Your opinion counts.

**Appendix B.** **Geographic distribution of physicians in the survey.**

|  | **Total Sample** |
| --- | --- |
|  | N=440 |
| *Physicians work in, n (%)* |  |
| Alabama | 12 (3) |
| Alaska | 2 (0) |
| Arizona | 7 (2) |
| Arkansas | 3 (1) |
| California | 44 (10) |
| Colorado | 7 (2) |
| Connecticut | 7 (2) |
| Delaware | 2 (0) |
| Florida | 32 (7) |
| Georgia | 10 (2) |
| Hawaii | 3 (1) |
| Idaho | 2 (0) |
| Illinois | 41 (9) |
| Indiana | 6 (1) |
| Iowa | 5 (1) |
| Kansas | 1 (0) |
| Kentucky | 4 (1) |
| Louisiana | 3 (1) |
| Maine | 2 (0) |
| Maryland | 8 (2) |
| Massachusetts | 7 (2) |
| Michigan | 17 (4) |
| Minnesota | 7 (2) |
| Mississippi | 2 (0) |
| Missouri | 9 (2) |
| Montana | 1 (0) |
| Nebraska | 2 (0) |
| Nevada | 3 (1) |
| New Hampshire | 2 (0) |
| New Jersey | 17 (4) |
| New Mexico | 2 (0) |
| New York | 34 (8) |
| North Carolina | 14 (3) |
| North Dakota | 0 (0) |
| Ohio | 18 (4) |
| Oklahoma | 4 (1) |
| Oregon | 5 (1) |
| Pennsylvania | 25 (6) |
| Rhode Island | 2 (0) |
| South Carolina | 6 (1) |
| South Dakota | 1 (0) |
| Tennessee | 10 (2) |
| Texas | 17 (4) |
| Utah | 3 (1) |
| Vermont | 1 (0) |
| Virginia | 14 (3) |
| Washington | 7 (2) |
| West Virginia | 2 (0) |
| Wisconsin | 6 (1) |
| Wyoming | 0 (0) |
| District of Columbia | 1 (0) |
